# Supplementary material for: Structural and Functional Strategies in Cenchrus Species to Combat Environmental Extremities Imposed by Multiple Abiotic Stresses
Source: Plants (Basel). 2024 Jan 11;13(2):203. doi: 10.3390/plants13020203 (PMC10818359; doi:10.3390/plants13020203)
Supplement: Supplementary file 1 [file plants-13-00203-s001.zip › plants-2594197-supplementary.pdf]

Supplementary Table S1a. Morphology, physiology and anatomy data of *Cenchrus pennisetiformis*

	Control	Drought	Salinity	Cold
Morphology				
Plant height (cm)	60.20±6.01	23.70±1.7	28.57±1.87	24.57±1.72
Root length (cm)	8.43±0.38	30.23±2.4	5.40±0.39	14.83±1.39
Shoot fresh weight (g plant^{-1})	17.15±1.52	31.07±2.5	8.06±0.63	19.17±1.54
Shoot dry weight (g plant^{-1})	7.06±0.36	12.54±0.10	2.86±1.37	7.81±0.63
Root fresh weight (g plant^{-1})	15.40±1.49	12.33±0.98	4.67±0.35	7.57±0.66
Root dry weight (g plant^{-1})	6.40±0.58	5.64±0.38	1.64±0.54	3.36±2.53
Leaf area (cm^2)	305.89±29.7	126.04±10.42	69.92±5.86	134.97±12. 65
Number of leaves per plant	42.43±3.6	70.37±5.87	36.33±2.85	34.50±3.16
Inflorescence length (cm)	3.55±0.18	4.53±0.37	3.50±0.25	16.70±1.43
Root anatomy				
Root radius (µm)	1366.38±129.7	932.12±89.7	569.93±4.980.	516.03±49.83
Epidermis thickness (µm)	69.05±5.57	76.31±6.81	0.00±0.00	0.00±0.00
Cortical cell area (µm^2)	2398.61±228.6	7040.19±699.11	805.88±78.3	5441.40±498.3
Endodermis thickness (µm)	41.79±3.95	41.79±3.82	34.52±2.91	38.16±2.94
Pericycle thickness (µm)	94.48±8.67	76.31±6.36	78.13±6.72	96.30±8.95
Metaxylem area (µm^2)	10896.64±1008.4	13446.57±1289.21	5193.24±4.98	5528.74±530.33
Phloem area (µm^2)	2399.48±230.5	2049.28±198.91	1275.40±123.4	371.81±30.4
Aerenchyma thickness (µm)	436.08±40.51	381.57±35.83	0.00±0.00	0.00±0.00
Pith thickness (µm)	968.46±90.21	675.92±6.58	570.54±53.2	380.96±36.32
Stem anatomy				
Stem radius (µm)	1331.86±129.32	1221.02±119.34	932.12±90.32	1188.32±116.54
Epidermis thickness (µm)	29.07±2.70	42.40±3.91	59.96±4.82	61.78±5.83
Cortical cell area (µm)	48257.55±4002.67	28785.05±2400.23	19543.40±1890.21	26865.47±2640.75
Metaxylem area (µm^2)	38.16±3.42	43.61±3.98	29.07±2.53	59.96±5.44
Vascular bundle area (µm^2)	24098.51±2399.21	35745.69±3489.85	32183.23±3256.36	30056.13±3001.78
Sclerenchyma thickness (µm)	129.01±10.8	99.94±8.92	94.48±8.93	148.99±12.90
Phloem area (µm^2)	3507.12±345.21	4021.61±399.21	5539.98±549.76	5028.95±487.54
Leaf sheath anatomy				
Leaf sheath thickness (µm)	290.72±2.81	1108.37±108.53	508.76±49.81	454.25±43.23
Epidermis thickness (µm)	22.41±1.80	54.51±5.43	27.26±2.50	39.97±3.65
Vascular bundle area (µm^2)	34414.09±3354.67	47384.23±4677.08	36921.65±3500.06	46606.02±4500.22
Sclerenchyma thickness (µm)	54.51±4.90	127.19±11.91	127.19±11.87	145.36±12.34
Parenchyma cell area (µm^2)	3980.97±388.04	49459.45±4876.04	5360.99±533.07	2734.10±271.06
Leaf blade anatomy				
Epidermis thickness (µm)	32.71±3.01	58.14±4.7	41.79±3.96	23.62±2.14
Parenchyma cell area (µm^2)	1718.98±167.87	2907.04±286.05	13579.73±1323.98	3168.17±313.65
Mesophyll thickness (µm)	96.30±94.03	132.64±11.80	127.19±11.6	93.88±9.23
Metaxylem area (µm^2)	2338.08±229.07	1814.95±178.04	2156.50±1978.54	1868.56±183.64
Phloem area (µm^2)	3838.30±379.08	2798.09±276.03	7015.98±698.48	2658.01±263.47
Vascular bundle area (µm^2)	28785.05±2788.96	94405.29±9456.37	99452.40±9800.76	45317.65±4501.08
Midrib thickness (µm)	981.18±95.02	1295.52±127.66	639.58±62.83	585.07±57.63
Lamina thickness (µm)	387.02±36.40	388.84±36.07	459.70±43.82	387.02±35.52
Bulliform area (µm^2)	44167.63±4375.04	14228.24±1400.55	52883.57±5175.45	63008.92±6299.64
Adaxial stomatal density per mm^2	72.50±6.51	81.90±7.92	131.60±12.5	123.50±11.41
Abaxial stomatal density per mm^2	77.50±7.57	87.20±8.45	122.50±11.4	99.00±8.70
Adaxial stomatal area (µm^2)	309.50±29.87	221.90±20.66	211.40±20.8	218.50±19.91
Abaxial stomatal area (µm^2)	216.40±20.4	188.60±17.55	216.20±20.08	222.90±21.46
Physiology				
Total soluble proteins (µg g^{-1} f w.)	270.70±26.50	2901.28±289.11	3266.48±324.54	2716.93±270.17
Total free amino acids (µg g^{-1} f w.)	2.74±0.21	7.42±0.73	14.84±1.39	4.03±0.38
Total soluble sugars (mg g^{-1} d w.)	16.72±1.43	26.23±2.44	20.93±1.98	16.70±1.56
Glycine betaine (µmol g^{-1} f w.)	33.25±3.11	52.51±4.91	37.51±3.64	50.04±4.98
Proline (µmol g^{-1} f w.)	75.15±7.42	250.27±2.12	311.10±31.44	183.56±17.34
Chlorophyll *a* (mg g^{-1} f w.)	1.17±0.01	2.00±0.2	1.41±0.11	1.00±0.10
Chlorophyll *b* (mg g^{-1} f w.)	0.70±0.006	1.10±0.01	0.83±0.07	0.63±0.06
Total chlorophyll (mg g^{-1} f w.)	1.87±0.16	3.10±0.33	2.25±0.24	1.64±0.15
Shoot Na^+ (mg g^{-1} f.w.)	18.50±1.74	30.50±2.98	65.50±6.43	20.10±1.90
Shoot K^+ (mg g^{-1} f.w.)	36.34±3.41	23.66±2.1	33.46±2.98	21.85±2.02
Shoot Ca^{2+} (mg g^{-1} f.w.)	7.53±0.76	8.20±0.8	8.85±0.87	8.99±0.87
Carotenoids (mg g^{-1} f w.)	0.04±0.004	0.03±0.003	0.06±0.006	0.03±0.002

Supplementary Table S1b. Morphology, physiology and anatomy data of *Cenchrus prieurii*

Morphology	Control	Drought	Salinity	Cold
Plant height (cm)				
Root length (cm)	17.00±1.60	20.00±0.18	19.00±1.62	21.00±0.19
Shoot fresh weight (g plant^{-1})	6.00±0.58	17.00±1.53	36.00±3.33	5.00±0.47
Shoot dry weight (g plant^{-1})	27.35±2.41	14.82±1.37	22.20±1.82	9.97±0.85
Root fresh weight (g plant^{-1})	10.55±1.04	5.69±0.48	8.62±0.84	3.75±0.29
Root dry weight (g plant^{-1})	8.20±0.75	10.90±1.01	6.30±0.54	25.33±2.31
Leaf area (cm^2)	3.36±0.29	4.24±0.37	2.77±019	11.50±0.98
Number of leaves per plant	36.99±3.57	40.10±3.82	205.72±19.4	49.98±4.64
Inflorescence length (cm)	32.00±2.91	29.00±2.51	55.00±4.83	35.00±3.32
Root anatomy				
Root radius (µm)	781.31±70.13	1277.96±125.32	890.33±7821	926.67±87.61
Epidermis thickness (µm)	55.12±4.72	55.12±4.81	27.26±2.11	45.43±3.92
Cortical cell area (µm^2)	3616.07±	3958.49±390.36	1980.11±193.42	1067.87±1000.56
Endodermis thickness (µm)	45.43±	55.12±4.72	54.51±4.98	36.95±2.90
Pericycle thickness (µm)	19.87±	50.88±4.91	19.81±1.83	22.35±1.88
Metaxylem area (µm^2)	8162.54±772.8	11018.56±1100.23	22789.39±2001.23	8108.06±790.0
Phloem area (µm^2)	1067.87±105.4	4172.06±387.64	3184.60±298.7	2397.75±200.15
Aerenchyma thickness (µm)	198.66±17.63	0.00±0.00	0.00±0.00	91.46±8.53
Pith thickness (µm)	278.61±25.3	926.67±89.23	763.14±70.23	744.97±68.45
Stem anatomy				
Stem radius (µm)	1285.22±120.65	1146.53±110.5	1221.02±120.32	986.03±90.54
Epidermis thickness (µm)	21.80±1.73	30.89±2.93	32.71±2.53	23.62±1.90
Cortical cell area (µm)	15690.41±1467.09	9207.07±888.20	7776.89±65.73	21244.22±2100.56
Metaxylem area (µm^2)	32.71±2.90	36.34±2.82	45.43±3.91	32.71±2.63
Vascular bundle area (µm^2)	20568.04±1998.03	35183.65±3401.65	38661.38±3777.21	30768.62±3002.45
Sclerenchyma thickness (µm)	58.14±5.32	109.02±9.74	145.36±12.62	72.68±6.72
Phloem area (µm^2)	7377.41±690.02	13482.02±1267.23	11252.89±1173.89	5174.22±501.23
Leaf sheath anatomy				
Leaf sheath thickness (µm)	617.78±59.36	563.27±54.33	368.25±34.7	999.35±95.6
Epidermis thickness (µm)	21.80±1.94	21.80±1.87	36.34±3.47	29.07±2.43
Vascular bundle area (µm^2)	35278.77±3467.89	31301.26±3003.55	45481.94±4400.03	79567.46±7850.05
Sclerenchyma thickness (µm)	63.60±5.91	72.68±68.98	54.51±5.21	90.85±8.91
Parenchyma cell area (µm^2)	11474.25±1009.63	15574.54±1408.75	2341.54±214.5	3150.88±301.77
Leaf blade anatomy				
Epidermis thickness (µm)	58.14±5.62	55.12±5.32	45.43±44.32	55.12±5.42
Parenchyma cell area (µm^2)	4753.99±473.89	2002.07±199.73	7846.93±780.65	5244.26±522.43
Mesophyll thickness (µm)	218.65±21.40	222.28±21.14	195.02±19.22	145.97±14.21
Metaxylem area (µm^2)	989.19±97.81	1458.71±143.65	1021.36±100.46	1083.44±106.55
Phloem area (µm^2)	4155.63±4002.58	7888.44±776.43	12583.63±1250.36	10106.33±9998.87
Vascular bundle area (µm^2)	25608.24±2498.43	54736.57±5300.65	39031.46±3897.04	44075.11±4401.44
Midrib thickness (µm)	436.69±42.67	963.62±95.43	645.64±63.42	473.03±46.32
Lamina thickness (µm)	327.67±31.43	490.59±48.06	393.68±39.4	327.67±31.75
Bulliform area (µm^2)	12516.18±1246.63	25086.84±2500.46	14023.31±1400.25	5235.61±516.57
Adaxial stomatal density per mm^2	73.80±7.26	75.80±7.23	65.30±6.31	80.40±7.90
Abaxial stomatal density per mm^2	77.80±7.54	82.50±7.91	66.80±6.55	79.40±7.84
Adaxial stomatal area (µm^2)	656.30±64.73	579.30±56.72	651.20±64.52	457.00±44.56
Abaxial stomatal area (µm^2)	644.30±63.62	536.30±53.54	548.00±50.86	455.80±43.57
Physiology				
Total soluble proteins (µg g^{-1} f w.)	2695.86±265.89	3068.96±305.96	2658.55±264.87	1585.33±157.43
Total free amino acids (µg g^{-1} f w.)	6.23±0.61	13.42±1.29	5.30±0.49	2.90±0.27
Total soluble sugars (mg g^{-1} d w.)	18.91±1.70	25.77±2.47	23.09±2.10	26.41±2.57
Glycine betaine (µmol g^{-1} f w.)	52.02±4.98	33.28±3.30	32.86±3.01	28.71±2.48
Proline (µmol g^{-1} f w.)	360.81±35.45	429.65±41.84	351.49±34.67	262.05±25.65
Chlorophyll *a* (mg g^{-1} f w.)	1.74±0.16	2.24±0.25	2.23±0.21	2.29±0.27
Chlorophyll *b* (mg g^{-1} f w.)	1.65±0.15	1.72±0.16	1.65±0.14	1.20±0.10
Total chlorophyll (mg g^{-1} f w.)	6.39±0.61	3.96±0.34	3.88±0.37	3.49±0.36
Shoot Na^+ (mg g^{-1} f.w.)	25.60±2.48	31.50±2.95	49.70±4.86	16.20±1.57
Shoot K^+ (mg g^{-1} f.w.)	22.42±2.01	28.71±2.65	32.57±3.42	21.81±1.96
Shoot Ca^{2+} (mg g^{-1} f.w.)	8.12±0.89	10.90±1.01	8.17±0.80	8.17±0.78
Carotenoids (mg g^{-1} f w.)	0.03±0.003	0.01±0.001	0.03±0.003	0.09±0.009

Supplementary Table S1c. Morphology, physiology and anatomy data of *Cenchrus setiger*

Morphology	Control	Drought	Salinity	Cold
Plant height (cm)				
Root length (cm)	26.00±2.42	37.00±	35.02±2.71	29.00±0.19
Shoot fresh weight (g plant^{-1})	6.09±0.58	9.09±	4.09±0.34	7.09±0.64
Shoot dry weight (g plant^{-1})	24.07±2.21	18.94±	18.03±1.65	23.59±1.96
Root fresh weight (g plant^{-1})	10.08±0.95	7.92±	6.98±0.57	9.87±0.89
Root dry weight (g plant^{-1})	9.67±0.86	8.30±	7.33±0.64	9.50±0.87
Leaf area (cm^2)	4.02±0.35	3.86±	3.92±0.31	4.33±0.35
Number of leaves per plant	122.14±11.3	249.16±	151.10±14.61	82.83±6.69
Inflorescence length (cm)	46.06±	66.00±0.53	66.00±5.82	42.00±3.81
Root anatomy				
Root radius (µm)	793.42±71.73	587.50±55.09	720.74±68.43	963.01±90.3
Epidermis thickness (µm)	36.95±3,12	53.90±5.21	20.59±1.82	22.41±1.92
Cortical cell area (µm^2)	475.57±44.52	1924.77±190.76	2390.83±231.80	3105.92±299.33
Endodermis thickness (µm)	36.34±3.13	36.34±3.22	29.07±2.11	50.88±4.53
Pericycle thickness (µm)	27.26±2,23	21.80±1.88	27.26±2.56	23.02±1.99
Metaxylem area (µm^2)	8740.14±85.42	9656.70±935.48	3638.56±350.21	8025.92±798.43
Phloem area (µm^2)	474.71±44.67	1599.65±150.82	1409.42±139.01	468.65±44.35
Aerenchyma thickness (µm)	0.00±0.00	90.85±8.91	145.36±12.43	54.51±4.65
Pith thickness (µm)	478.48±40.43	236.21±20.22	68.44±5.53	563.27±50.23
Stem anatomy				
Stem radius (µm)	781.92±75.32	781.92±72.65	781.31±76.23	771.62±75.23
Epidermis thickness (µm)	44.82±3.36	19.99±1.53	27.26±2.56	72.68±69.80
Cortical cell area (µm)	7049.70±703.67	1337.65±130.66	6077.81±600.09	13732.78±1290.66
Metaxylem area (µm^2)	36.34±3.02	90.85±8.90	36.95±2.99	46.03±4.23
Vascular bundle area (µm^2)	31429.23±3001.43	5198.43±501.43	23380.83±2290.89	22862.03±2190.38
Sclerenchyma thickness (µm)	62.99	36.34±3.01	59.36±4.83	90.85±8.80
Phloem area (µm^2)	7119.74±699.09	592.30±56.70	804.15±78.90	4906.17±487.6
Leaf sheath anatomy				
Leaf sheath thickness (µm)	218.04±19.98	744.97±73.52	290.72±28.51	726.80±70.54
Epidermis thickness (µm)	23.62±2.01	21.80±1.90	21.80±2.01	27.26±2.5.23
Vascular bundle area (µm^2)	9331.58±927.88	24065.65±2387.33	6450.48±639.80	14533.47±1440.36
Sclerenchyma thickness (µm)	36.34±3.31	36.34±3.42	27.26±2.604	36.34±3.21
Parenchyma cell area (µm^2)	3897.96±385.77	7789.00±769.09	4157.36±412.66	5846.94±570.33
Leaf blade anatomy				
Epidermis thickness (µm)	27.26±2.62	26.65±25.55	20.59±19.54	27.26±26.5
Parenchyma cell area (µm^2)	14542.12±1447.85	345.01±33.45	7252.90±723.87	2713.35±270.66
Mesophyll thickness (µm)	205.93±19.07	109.02±10.43	90.85±8.91	81.77±8.03
Metaxylem area (µm^2)	1117.16±110.76	302.64±30.31	316.47±31.43	313.01±29.33
Phloem area (µm^2)	4549.92±450.78	1286.64±125.75	4212.70±420.35	2338.08±233.76
Vascular bundle area (µm^2)	23294.36±2300.96	8565.48±850.55	12968.41±1280.65	11818.39±1177.47
Midrib thickness (µm)	944.84±93.31	218.04±20.84	690.46±68.00	290.72±28.00
Lamina thickness (µm)	375.51±35.43	199.87±18.77	278.61±27.58	290.72±28.75
Bulliform area (µm^2)	4662.33±463.22	1174.23±116.44	1566.79±154.57	2345.00±232.65
Adaxial stomatal density per mm^2	52.70±5.01	78.20±7.64	75.40±7.32	81.90±8.00
Abaxial stomatal density per mm^2	54.10±5.32	69.00±6.54	77.40±7.43	83.10±7.91
Adaxial stomatal area (µm^2)	407.60±39.74	254.60±24.43	302.80±29.04	267.80±26.45
Abaxial stomatal area (µm^2)	339.60±33.84	210.50±20.13	236.70±23.55	155.40±15.42
Physiology				
Total soluble proteins (µg g^{-1} f w.)	277.28±26.45	2475.51±245.73	1582.26±156.74	2631.33±262.00
Total free amino acids (µg g^{-1} f w.)	6.99±0.65	7.42±0.72	5.62±0.49	6.66±0.64
Total soluble sugars (mg g^{-1} d w.)	27.31±2.61	30.66±2.97	26.94±2.56	29.62±2.84
Glycine betaine (µmol g^{-1} f w.)	26.30±2.57	29.55±2.65	30.21±3.00	34.02±3.40
Proline (µmol g^{-1} f w.)	158.54±15.4	317.48±30.45	260.57±25.01	266.46±25.7
Chlorophyll *a* (mg g^{-1} f w.)	1.68±0.15	1.69±0.14	1.42±0.12	1.48±0.13
Chlorophyll *b* (mg g^{-1} f w.)	1.15±0.10	1.23±0.11	1.17±0.14	0.90±0.009
Total chlorophyll (mg g^{-1} f w.)	2.83±0.26	2.93±0.26	2.59±0.23	2.38±0.21
Shoot Na^+ (mg g^{-1} f.w.)	19.50±1.86	35.20±3.53	68.70±6.52	29.80±2.85
Shoot K^+ (mg g^{-1} f.w.)	30.63±3.01	27.22±2.65	24.81±2.31	27.82±2.65
Shoot Ca^{2+} (mg g^{-1} f.w.)	7.55±0.73	10.67±1.00	8.93±0.83	9.89±0.93
Carotenoids (mg g^{-1} f w.)	0.04±0.004	0.05±0.005	0.03±0.003	0.07±0.003
